# Functional analysis of Cti6 core domain responsible for recruitment of epigenetic regulators Sin3, Cyc8 and Tup1

**DOI:** 10.1007/s00294-020-01109-4

**Published:** 2020-09-26

**Authors:** Rasha Aref, Hans-Joachim Schüller

**Affiliations:** 1 Department of Genetics, Faculty of Agriculture, Ain Shams University, Shoubra El-Khaymah, Cairo, 11241 Egypt; 2 Center for Functional Genomics of Microbes, Abteilung Molekulare Genetik Und Infektionsbiologie, Felix-Hausdorff-Straße 8, 17487 Greifswald, Germany

**Keywords:** Histone deacetylase Rpd3, Corepressor Sin3, Cti6, Protein–protein interaction

## Abstract

Mapping of effective protein domains is a demanding stride to disclose the functional relationship between regulatory complexes. Domain analysis of protein interactions is requisite for understanding the pleiotropic responses of the respective partners. Cti6 is a multifunctional regulator for which we could show recruitment of co-repressors Sin3, Cyc8 and Tup1. However, the responsible core domain tethering Cti6 to these co-repressors is poorly understood. Here, we report the pivotal domain of Cti6 that is indispensable for co-repressor recruitment. We substantiated that amino acids 450–506 of Cti6 bind PAH2 of Sin3. To analyse this Cti6–Sin3 Interaction Domain (CSID) in more detail, selected amino acids within CSID were replaced by alanine. It is revealed that hydrophobic amino acids V467, L481 and L491 L492 L493 are important for Cti6–Sin3 binding. In addition to PAH2 of Sin3, CSID also binds to tetratricopeptide repeats (TPR) of Cyc8. Indeed, we could demonstrate Cti6 recruitment to promoters of genes, such as *RNR3* and *SMF3*, containing iron-responsive elements (IRE). Importantly, Sin3 is also recruited to these promoters but only in the presence of functional Cti6. Our findings provide novel insights toward the critical interaction domain in the co-regulator Cti6, which is a component of regulatory complexes that are closely related to chromatin architecture and the epigenetic status of genes that are regulated by pleiotropic co-repressors.

**Electronic supplementary material:**

The online version of this article (10.1007/s00294-020-01109-4) contains supplementary material, which is available to authorized users.

## Introduction

The functional associations and affinities that take place between proteins are at the essence of numerous regulatory processes and their structural principle helps to provide context in molecular function. Identifying domains within regulatory protein complexes are a prerequisite step for devising protein structure and functional annotation. Transcriptional activity of genes is governed by regulatory complexes that assemble/disassemble on the promoter region and control the chromatin architecture (Lemon and Tjian 2000; Cairns 2009). In eukaryotic systems, Rpd3 histone deacetylase addresses defined control regions in association with different co-repressor complexes (Kadosh and Struhl 1998). In yeast, one of the associated subunits of the Rpd3L histone deacetylase complex is the regulatory protein Cti6, implicated in many regulatory pathways (Papamichos-Chronakis et al. 2002; Puig et al. 2004). In most if not all targeted cellular functions, Cti6 fulfills the respective function associated with transcriptional regulatory complexes. Among these regulatory complexes that play pivotal roles in yeast cell biology are Cyc8-Tup1 (Váchová and Palková 2019) and Sin3 (Adams et al. 2018) complexes. Both co-repressors include but are not limited to the following features: (1) contain domains for multiple protein interactions via paired amphipathic helices (PAH) and tetratricopeptide repeat (TPR) motifs in Sin3 and Cyc8, respectively (Wang et al. 1990; Tzamarias and Struhl 1994); (2) do not encode DNA-binding proteins but are rather targeted to selected promoters through regulatory proteins (Kliewe et al. 2020; Davie et al. 2003) which in turn regulates transcription; (3) implicated in numerous regulatory pathways (Maqani et al. 2018; Chaubal and Pile 2018), thus conferring pleiotropic properties to these co-repressors; (4) could perform dual sides of gene transcription control either positively or negatively by configuring chromatin architecture based on its histone deacetylase activity (Adams et al. 2018; Davie et al. 2003; Papamichos-Chronakis et al. 2002). Strikingly, Cti6 (Cyc8—Tup1 Interacting Protein 6) interacts with Sin3 and Cyc8 co-repressor complexes independently (Papamichos-Chronakis et al. 2002; Puig et al. 2004). Cti6 has been initially identified as a positive regulator as it interacts with SAGA coactivator complex and thence activates transcription of target genes (Papamichos-Chronakis et al. 2002). Subsequent studies showed that Cti6 is a subunit of the Rpd3L histone deacetylase complex (Puig et al. 2004; Carrozza et al. 2005), which indicates that Cti6 acts as a repressor as well. Another striking feature of Cti6 is that it can bind more than one regulatory protein altogether, such as Cyc8/Tup1 and SAGA complex (Papamichos-Chronakis et al. 2002). Various studies reported the involvement of Cti6 in numerous physiological pathways, such as transcriptional de-repression of *GAL1* and *ANB1*, which respond to diverse signals (low glucose and oxygen, respectively). In addition, Cti6 is important for iron metabolism in yeast (Puig et al. 2004). Cti6 has a differential role for certain iron-regulated genes, such as *ARN1*, *FET3*, *SMF3* and *RNR3* (Kaplan et al. 2006; Puig et al. 2004). Interestingly, all detected Cti6 regulatory functions have been fulfilled through association either with Sin3/Rpd3 or Cyc8/Tup1 co-repressor complexes (Papamichos-Chronakis et al. 2002; Puig et al. 2004). Although Cti6 mediates interaction with many regulatory complexes, the pivotal domain of Cti6 that leads to the recruitment of Sin3 or Cyc8 co-repressors has not yet been investigated. In addition, no analysis has been carried out so far regarding the required characteristics of such a domain to ensure Cti6 function in the respective cellular pathways.

Therefore, it was worthy to extend the scope on the regulatory protein Cti6 by characterizing extensively its interaction with both co-repressors and explore additional aspects regarding particular Cti6 target genes. In this report, we show that Cti6 not only interacts directly with both Sin3 and Cyc8/Tup1 co-repressors but also identify precisely the minimal interaction domain responsible for binding of all tested co-repressors. Our in vitro interaction studies showed that amino acids 450–506 of Cti6 bind PAH2 of Sin3 and the same domain mediates the interaction with Cyc8 co-repressor as well. To analyse Cti6–Sin3 interaction domain (CSID) in more detail, selected amino acids within CSID were replaced by alanine. It turned out that hydrophobic amino acids V467, L481 and at least one of the lysines at the positions 491–493 is important for Cti6–Sin3 binding. The results of this work also suggest that repression is not executed entirely via Sin3, but rather CSID is also important for contacting pleiotropic co-repressor Cyc8 via its tetratricopeptide repeats (TPR). Chromatin immunoprecipitation (ChIP) analyses demonstrated Cti6 recruitment to promoters of genes, such as *RNR3* and *SMF3*, containing iron-responsive elements (IRE). Importantly, Sin3 was also recruited to these promoters but only in the presence of functional Cti6.

## Materials and methods

### Yeast strains and media

According to the various experimental purposes, various strains of the yeast *Saccharomyces cerevisiae* were used. All of the following strains are haploid. *S. cerevisiae* strain C13-ABY.S86 lacking four vacuolar proteinases (*pra1 prb1 prc1 cps1*; De Antoni and Gallwitz 2000) was used for protein–protein interaction assays. Strains used for ChIP analyses were derived from C13-ABY.S86 by gene replacement experiments (construction of epitope-tagged variant of *CTI6* at its authentic chromosomal positions; see below) or gene disruptions (introduction of deletion mutant allele for *cti6*). To obtain such isogenic null mutant, disruption plasmid pRAR81 (*ΔCTI6::LEU2*) was used. Complete genotypes of all strains used are available as supporting online Table 1 (section a). For ChIP analysis, the investigated strains were cultivated under respective repression conditions as described in Puig et al. (2004).

### In vitro interaction assays (GST pull-down)

GST- and HA-tagged proteins used for interaction assays by affinity chromatography were synthesized by *E. coli* strain BL21 (Stratagene/Agilent). The tac promoter controlling GST fusion genes was induced with 1 mM IPTG. Similarly, tetR-dependent gene expression was activated by 0.2 mg/l anhydrotetracycline. Derepression of *MET25*-dependent gene fusions was achieved by cultivating yeast transformants in the absence of methionine. GST fusion proteins synthesized in *E. coli* were released by sonication, immobilized on glutathione (GSH) Sepharose and subsequently incubated with yeast or bacterial total protein extracts containing HA fusions. To avoid unspecific interactions, protein extracts were pre-cleared by treatment with GSH Sepharose beads prior to incubation with GST fusions. Details on washing steps at intermediary stringency have been described (Wagner et al. 2001). After release of GST fusions with free GSH (10 mM), eluates were separated by SDS/PAGE and proteins transferred to a filter. Following incubation with anti-HA-peroxidase conjugate, HA fusion proteins were detected with POD chemiluminescent substrate (antibody conjugate and substrate from Roche Biochemicals).

### Two-hybrid assays

To perform two-hybrid assays, strain PJ69-4A was used (*MATa trp1-901 leu2-3,112 ura3-52 his3-200 gal4Δ gal80Δ UAS*_*GAL2*_*-ADE2 LYS2::UAS*_*GAL1*_*-HIS3 met2::UAS*_*GAL7*_*-lacZ*; James et al. 1996). DNA fragments encoding interaction domains of Sin3 (PAH1 & PAH2) were inserted into plasmids pGBD-C1 (2 µm GAL4_DBD_ *TRP1*) while Cti6 domains (aa 351–506; aa 430–506; aa 450–506) were inserted in pGAD-C1 (2 µm GAL4_TAD_ *LEU2*). Double-transformed strains containing both types of fusion plasmids were first selected on medium lacking leucine and tryptophan (-L-T) and subsequently transferred to medium devoid of adenine (-L-T-A).

### Site-directed mutagenesis

To alter selected residues in the coding region of Cti6, the QuikChange site-directed mutagenesis kit of Stratagene was used. To obtain mutations within CSID, plasmid pRAR47 containing the Cti6 coding region was used. To replace selected residues against alanine, we used pairs of mutagenic primers introducing a GCA codon instead of the natural codon, flanked by 15–19 nucleotides on both the sides. DNA sequencing was used to confirm the presence of the desired mutant alleles of *cti6* (V467A, L481A and L491A L492A L493A) and the absence of any other change in the plasmids obtained (pRAR20, pRAR37, pRAR49, pRAR65-67).

### Chromatin immunoprecipitation

Essentially, chromatin immunoprecipitation (ChIP) analysis followed the procedure described by Cobb and van Attikum (2010). Chromosomal locus *CTI6* was modified such that it expressed a His-tagged Cti6 without alteration of gene copy number or control region. Tagging was performed by transformation of strain C13-ABY.S86 with a gene-specific modification fragment and selection for resistance against geneticin. The modification fragment was amplified by PCR, using gene-specific primers and plasmid pU6H3HA as a template (contains a His6-HA3-kanMX cassette; De Antoni and Gallwitz 2000). A strain which encodes epitope-tagged Sin3 (FKY11) was kindly provided by F. Kliewe; a *CTI6* gene deletion was introduced into FKY11 strain. The resulting strains RAY1 (*CTI6-HIS*_*6*_*-HA*_*3*_*-kanMX*), FKH11 (*SIN3-HIS*_*6*_*-HA*_*3*_*-kanMX*) and its isogenic *cti6* derivative RAY3 (*Δcti6 SIN3-HIS*_*6*_*-HA*_*3*_*-kanMX)* grew until mid-log phase and were treated with formaldehyde for 15 min. The cross-linking reaction was subsequently quenched for 5 min by addition of glycine to a final concentration of 125 mM. After lysis, cells were sonicated five times for 30 s to shear chromatin, using a Bandelin Sonoplus UW 70 microtip (35% power). After sonication, lysates were centrifuged for 10 min at 16,000 g to remove insoluble material and incubated for at least 4 h with His-Tag Dynabeads^®^ (Invitrogen/Dynal^®^). After elution of affinity-purified proteins and bound DNA with a buffer containing 300 mM imidazole, cross-linking was reversed by heating to 65 °C overnight. DNA was recovered and analyzed by PCR (29 amplification cycles), using specific primers against promoters of *RNR3* (− 400/− 100) and *SMF3* (− 350/− 80) or *ACT1* gene (+ 841/ + 1165) as a control.

### Plasmid constructions

To perform interaction assays, *Escherichia coli* expression plasmids (derived from pGEX-2TK; GE Healthcare) encoding various glutathione S-transferase (GST) fusions were constructed. Length variants of coding regions of *CTI6* gene were amplified by PCR and fused behind GST. Similarly, HA-tagged length variants of Sin3 representing PAH domains, Cyc8 and Tup1 were expressed in yeast using plasmid p426-MET25HA (Mumberg et al. 1994). For bacterial expression of selected Sin3 variants, plasmid pASK-IBA5 (tetR-regulated; IBA, Göttingen, Germany) was used. Yeast expression plasmid pCW117 used for synthesis of HA_3_-tagged Sin3 (full-length) has been described (Wagner et al. 2001). For bacterial synthesis of epitope-tagged Sin3, Cyc8 and Tup1 plasmids pSW11 (HA_3_-*SIN3*; full-length), pFK77 (HA_3_-*CYC8*; encoding aa 1–398 representing the TPR-containing domain) and pRAR110 (HA_3_-*TUP1*; full-length) derived from pASK-IBA5 (tetR-dependent; IBA, Göttingen, Germany) were used. To confirm authenticity of gene fragments obtained by PCR, GST fusions which encode minimal length variants of Cti6 were verified by DNA sequencing (LGC Genomics, Berlin, Germany). Plasmid names and fused sequences are mentioned in legends of figures and are described in detail in supporting online Table 1 (section b). Gene-specific primers used for PCR amplifications are available as supporting online Table 1 (section c). Plasmid pRAR81 was constructed by established procedures to disrupt the *CTI6* gene (*Δcti6::LEU2*). To construct this plasmid, flanking sequences upstream and downstream of the respective coding regions were amplified by PCR and inserted on both sides of the selection marker, allowing total deletion of the *CTI6* reading frame.

### Miscellaneous procedures

Transformation of *S. cerevisiae* strains, selection for yeast transformants on the respective synthetic media, PCR amplification and β-galactosidase assays have been described (Schwank et al. 1995; Wagner et al. 2001).

## Results

### Cti6 interacts directly with the pleiotropic co-repressor Sin3

Large-scale approaches, such as tandem affinity purification, have been used to analyze multiprotein complexes in *S. cerevisiae* (Gavin et al. 2002; Ho et al. 2002). Interestingly, Cti6 protein was independently identified when Rpd3 and Sin3 were affinity-purified using the tandem affinity purification epitope. Both Rpd3 and Sin3 copurified with Cti6 when the Ume1-Flag transcription factor was immunoprecipitated with anti-FLAG antibody (Ho et al. 2002).

Puig et al. (2004) have proved the functional relationship between Cti6 and Sin3, which suggests that these proteins might interact with one another. In support of this hypothesis, we used affinity chromatography as a suitable technique for demonstrating interaction in vitro. A glutathione S-transferase (GST)-Cti6 fusion protein (amino acids 1–506, comprising full length) was synthesized in *Escherichia coli* and subsequently bound to glutathione (GSH) Sepharose. A protein extract from *S. cerevisiae* (HA-Sin3 full length) was added to this affinity matrix. After intensive washing with increasing stringency, specifically bound protein was eluted by the addition of free GSH.

**Fig. 1:**
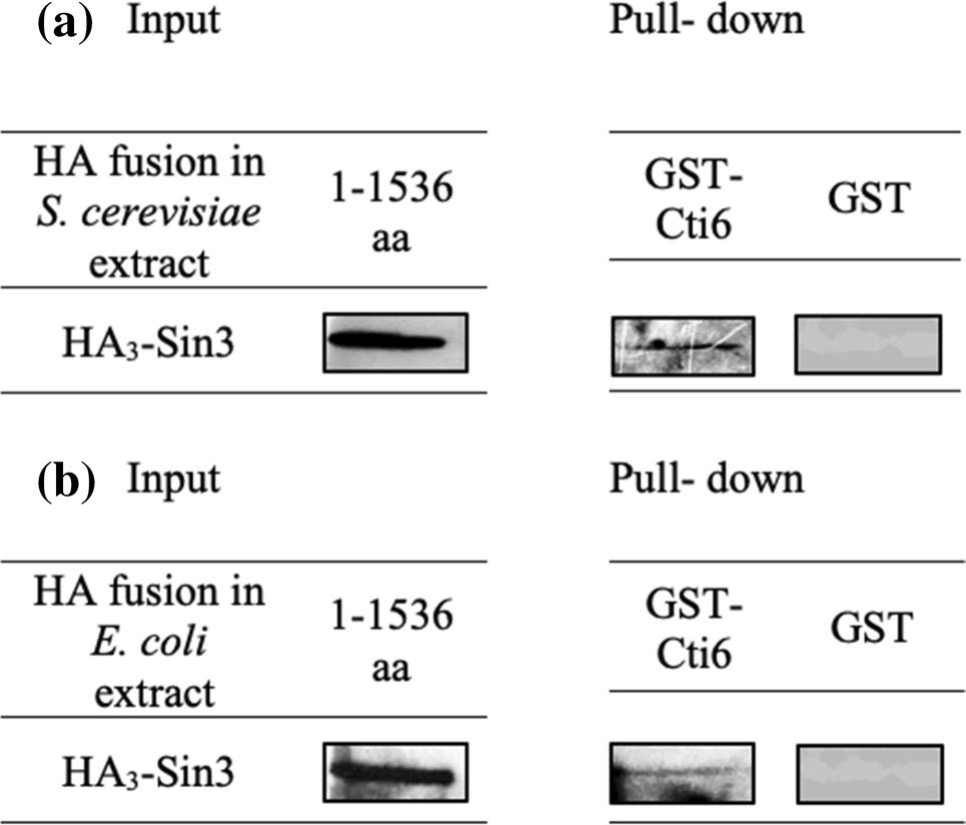
In vitro interaction of Cti6 with Sin3 shown by affinity chromatography. ** a** Full length of Cti6 was fused with GST, immobilized on GSH Sepharose and incubated with protein extract from yeast transformants containing full-length HA_3_-Sin3 synthesized by *S. cerevisiae* (strain C13-ABY.S86, plasmid pCW117). ** b** Bacterially synthesized HA_3_-Sin3 (*E.coli* strain BL21, plasmid pSW11) was incubated with GST-Cti6 (full-length fusion protein) bound to GSH Sepharose. GST-Cti6 fusion is encoded by plasmid pRAR3 (aa 1–506), GST vector was used as a negative control. Extracts containing 75 µg of total protein were analyzed for the input control. To achieve comparable amounts of HA-Sin3 for the interaction assay, total protein was adjusted accordingly

As is shown in Fig. 1a by immunodetection with anti-HA-antibody, the 175 kDa protein HA_3_-Sin3 could be bound by GST–Cti6 under stringent conditions but not by GST. Thus, Cti6 may execute its function by recruiting the general co-repressor Sin3. As interaction experiments performed with protein extracts from yeast cannot completely rule out indirect interactions mediated by distinct factors, HA_3_-Sin3 was thereafter also synthesized in *E. coli*. Since bacterial protein extracts should not contain yeast-specific factors, a direct interaction can be concluded by the use of HA_3_-Sin3 from *E. coli*. As is apparent from Fig. 1b, identical results were indeed obtained with extracts from *E. coli*, indicating that interaction between Cti6 and Sin3 occurs directly.

### PAH1 and PAH2 of Sin3 contact Cti6 in vitro

**Fig. 2:**
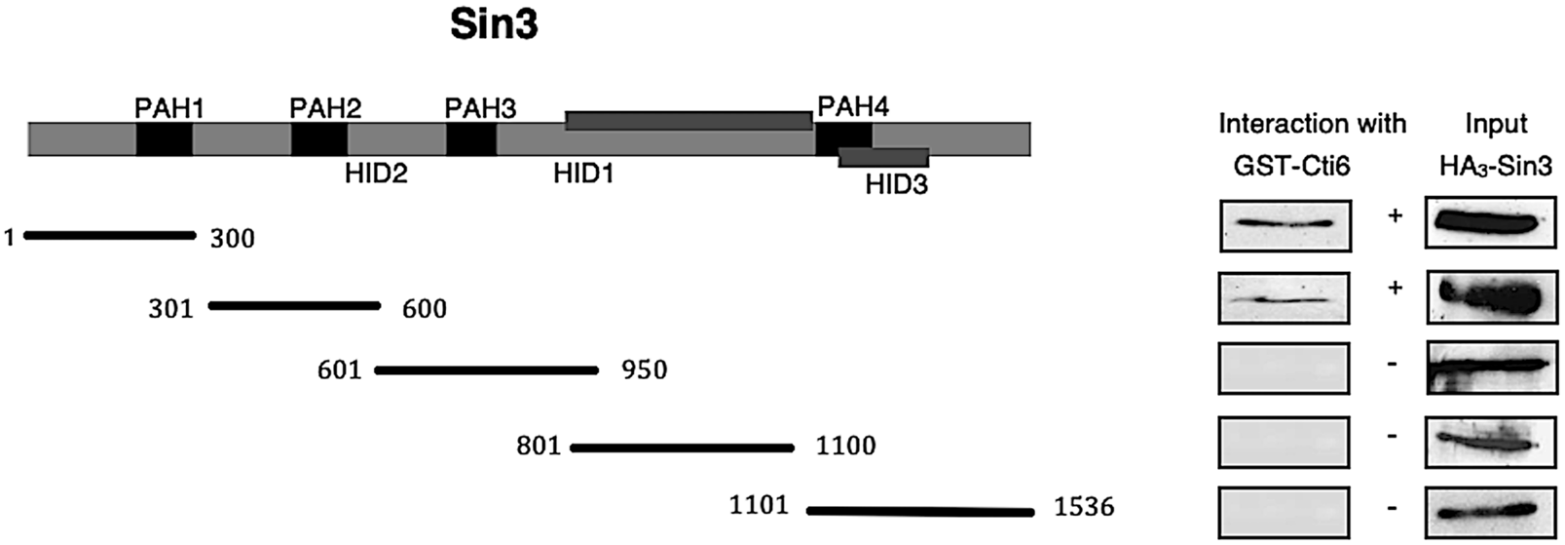
Mapping of Sin3 domains interacting with Cti6. GST-Cti6 fusion plasmid pRAR3 was used to synthesize full length of Cti6. The following expression plasmids representing individual PAH domains were used for synthesis of HA-tagged Sin3 length variants in *S. cerevisiae*: pCW83 (aa 1–300), pYJ91 (aa 301–600), pYJ90 (aa 601–950), pYJ89 (aa 801–1100) and pMP20 (aa 1101–1536). For input controls (shown in the right panel of the figure), 20% of protein used for the interaction assay was analyzed. PAH1-4: paired amphipathic helices 1–4

We, thus, wished to establish a physical map of interacting domains within Sin3 and Cti6. Sin3 contains four PAH motifs, which have been proposed to mediate various protein–protein interactions (Wang and Stillman 1993). Therefore, a GST–Cti6 fusion immobilized on GSH sepharose was incubated with HA-Sin3 length variants from yeast, representing individual structural and functional domains (PAH1-PAH4, HID). As can be seen in Fig. 2, amino acids 1–300 and 301–600 of Sin3 comprising its domains PAH1 and PAH2, respectively, are sufficient for interaction with Cti6. No interaction was detected with constructs representing C-terminal sequences of Sin3.

### CSID identified as the Cti6–Sin3 interaction domain

**Fig. 3:**
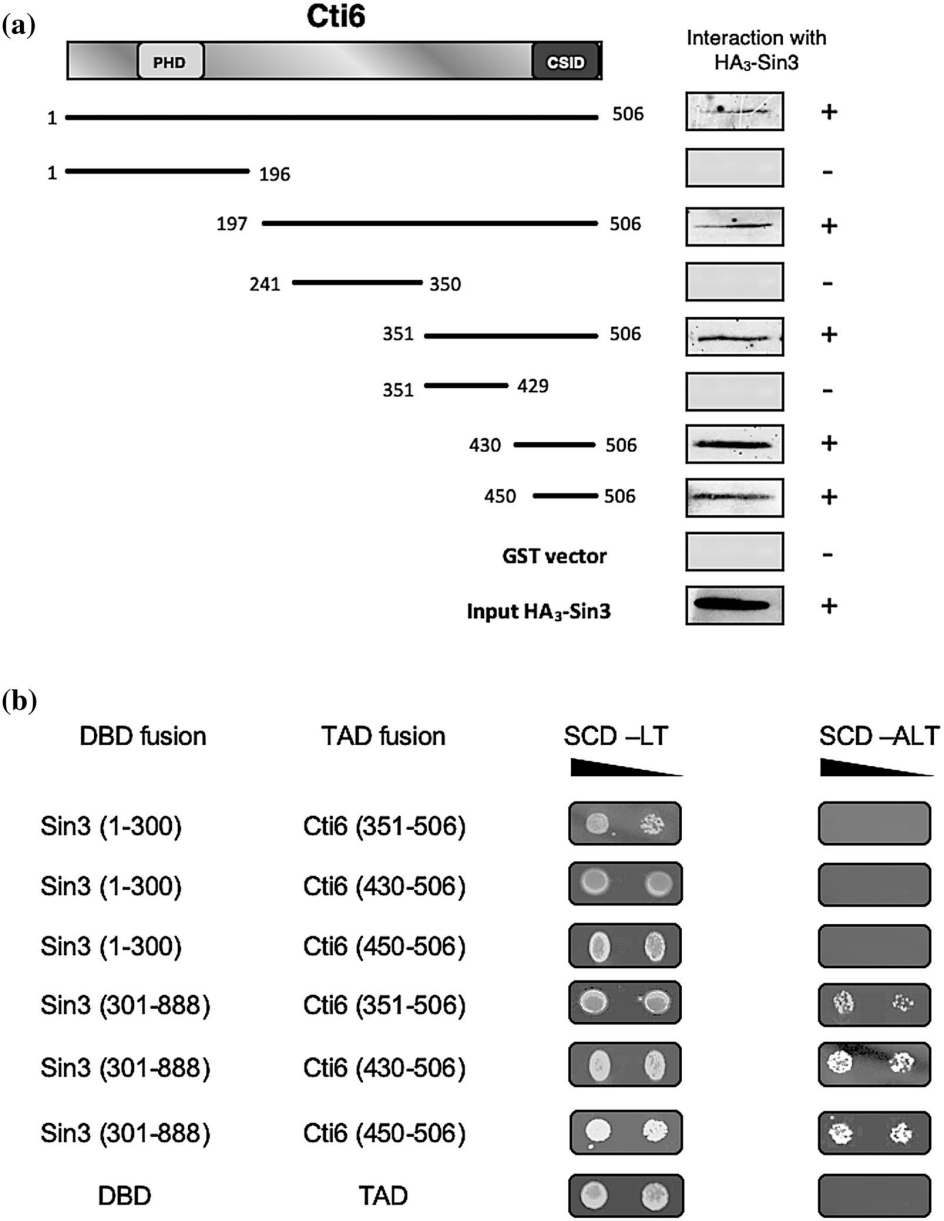
**a** Mapping of the Cti6 domain responsible for interaction with Sin3. Length variants of Cti6 were fused with GST, immobilized on GSH Sepharose and incubated with full-length HA_3_-Sin3 in total protein extract, synthesized by *S. cerevisiae* (Strain C13-ABY.S86, plasmid pCW117). GST-Cti6 fusions are encoded by plasmids pRAR3 (aa 1–506), pRAR10 (aa 1–196), pRAR11 (aa 197–506), pRAR14 (aa 241–350), pRAR15 (aa 351–506), pRAR30 (aa 351–429), pRAR31 (aa 430–506) and pRAR47 (aa 450–506). GST vector was used as a negative control. Input control is shown at the bottom of the figure (20% of protein used for the interaction assay). CSID: Cti6-Sin3 interaction domain; PHD: plant homeodomain. ** b** Interaction of Sin3 domains with various Cti6 domains shown by two-hybrid assay. Plasmids pWJ6 (aa 1–300) and pJW50 (aa 301–888) encoding the Gal4 DNA-binding domain (DBD) fused with Sin3 domains PAH1 and PAH2, respectively, were transformed into strain PJ69-4A, containing a *GAL2-ADE2* fusion (selection marker: *TRP1*). Correspondingly, various plasmids encoding fusions of Gal4 transcriptional-activation domain (TAD) with Cti6 were co-transformed (selection marker: *LEU2*): pRAR20 (Cti6, aa 351–506), pRAR37 (Cti6, aa 430–506), pRAR49 (Cti6, aa 450–506). As a negative control, empty pGBD-C1 and pGAD-C1 vectors were used. Growth in the absence of adenine is possible when a functional Gal4 activator is reconstituted by Cti6-Sin3 interaction in vivo. Selection plates (SCD-LT, absence of leucine and tryptophan; SCD-ALT, absence of leucine, tryptophan and adenine) were incubated for 48 h

Vice versa, we also mapped Cti6 domains required for Sin3 interaction. GST fusions of Cti6 length variants were immobilized and subsequently incubated with HA-tagged Sin3, synthesized in *S. cerevisiae*. As is apparent from Fig. 3a, the plant homeodomain (PHD) of Cti6 was dispensable for its interaction with Sin3. Instead, a domain of 56 amino acids in the C-terminus turned out as the CSID (residues 450–506), which is able to interact with PAH1 and PAH2 of Sin3.

To confirm our in vitro results obtained by GST pull-down, we used two hybrid analyses as a suitable technique for demonstrating interaction in vivo. Length variants of Sin3 comprising PAH1 (aa 1–300) and PAH2 (aa 301–888) were fused with the DNA-binding domain (DBD) of Gal4. Binding domains of Cti6 which have been shown to bind Sin3 in vitro were fused with Gal4 transcriptional-activation domain (TAD). Sin3–Cti6 interactions in vivo should reconstitute a functional Gal4 activator being able to stimulate expression of the *GAL2-ADE2* reporter gene of the recipient strain. As a negative control, empty vectors containing DBD and TAD were used.

As is shown in Fig. 3b, the negative control expectedly showed no growth on adenine-free medium. In contrast, co-transformation of DBD-Sin3 (aa 301–888) with TAD fused to Cti6 length variants restored growth on adenine-free medium (Fig. 3b), which is consistent with results from the in vitro analysis that proved interaction between Cti6 and the PAH2 of Sin3. Importantly, Cti6_450-506_ minimal interaction domain which has displayed in vitro interaction with PAH2 of Sin3 is able to show in vivo binding as well.

In contrast to in vitro results, DBD fusions of Sin3 (aa 1–300, containing PAH1) in combination with TAD fusions of Cti6 were unable to mediate growth on medium lacking adenine. Presumably, formation of functional interaction domains in vivo is prevented with certain length variants due to failure of correct protein folding. In summary, use of the "two-hybrid" system confirmed in vivo interaction between Cti6 and PAH2 of Sin3.

### Structural pattern within CSID

**Fig. 4:**
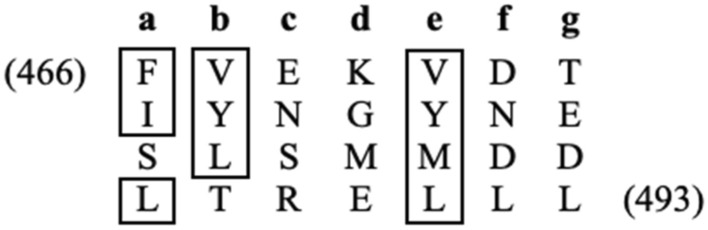
Amphipathic pattern of hydrophobic amino acids within CSID. Amino acids 466–493 of Cti6 are displayed as a heptad repeat (**a**–**g**). Hydrophobic residues at positions **a**, **b** and **e** are boxed

To define the structural principles of Cti6–Sin3 interaction, we looked for an amphipathic pattern of amino acids within a putative α-helix of CSID. Indeed, amino acids 466–493 form such a regular pattern of hydrophobic residues (apparent from the heptad display of the Cti6 sequence shown in Fig. 4).

### A lexA-Cti6 fusion protein mediates transcriptional repression

Cti6 can functionally associate in vivo with the Rpd3–Sin3 complex in *S. cerevisiae* (Puig et al. 2004), in addition to our results in this report, which definitely confirmed the direct interaction between Sin3 and Cti6. According to these findings, the assumption that Cti6 may act as a repressor is obvious. To test for repressor function, two strains containing integrated reporter genes (*CYC1-lacZ* without lexA-binding site; *CYC1-lacZ* with four lexA-binding sites) have been used. To quantify the repression effect of Cti6 in vivo, an effector plasmid was constructed, carrying the DNA-binding domain of the bacterial lexA repressor (lexA_DBD_) together with the Cti6 reading frame. The empty lexA plasmid (pRT-lexA) was used as a control.

If this protein actually acts as a repressor, gene activation should be reduced as a result of co-repressor recruitment (such as Sin3–HDAC), leading to inaccessible chromatin. As shown in Table 1, the tested repressor Cti6 indeed conferred a significant reduction of the reporter gene expression in the presence of lexA-binding sites. Recruitment of Cti6 to lexA-binding sites reduced the specific β-galactosidase activity more than fivefold (9 U/mg vs. 50 U/mg in the strain without lexA-binding sites). Our results show that Cti6 mediates transcriptional repression when recruited to a promoter. This is in accordance with the findings of Puig et al. (2004) who have shown the repression function of Cti6 when targeted to promoter associated with Rpd3 HDAC.

**Table 1:** Influence of Cti6 repressor fusion plasmid on the expression of the lexA_Op_-containing reporter gene

Plasmids	Specific β-galactosidase activity (U/mg)		Repression-factor
	NKTS (0 × lexA)	RTS (4 × lexA)	
pRAR27	**50** (8)	**9** (2)	5.6
pRT	**51** (14)	**47** (19)	1

Both *S. cerevisiae* reporter strains NKTS (without LexA binding sites) and RTS + lexA (4 LexA binding sites) were individually transformed with pRAR27 (LexA-Cti6) and pRT (empty LexA vector) and grown in SCD-Ura-Leu liquid medium to mid- log growth phase. After cell harvesting, the specific β-galactosidase activity was determined in crude extracts of the transformants. Specific β-galactosidase activities are given in nmol ONPG hydrolyzed per min per mg of protein (U/mg). Each experiment represents the mean value (in bold) of 5 independent strain cultivations and enzyme assays. ± *SD* standard deviation. The respective standard deviation is given in parenthesis

### Mutational analysis of Cti6 domain interacting with Sin3

As shown above, the C-terminus of Cti6 (residues 450–506) is able to interact with PAH2 of Sin3 in vitro and in vivo. Within this domain (designated CSID), an amphipathic pattern of hydrophobic amino acids could be identified (residues 466–493; Fig. 4). To investigate the possible importance of these residues for interaction with Sin3 (and consequently for regulated expression of Cti6 target genes), we performed a site-directed mutagenesis at selected positions leading to replacement of hydrophobic amino acids to alanine (single mutations V467A and L481A, triple mutation L491A L492A L493A). The influence of alanine substitutions in domains of Cti6 (aa 450–506) on in vitro interaction with Sin3 was examined by affinity chromatography ("GST pull-down").

**Fig. 5:**
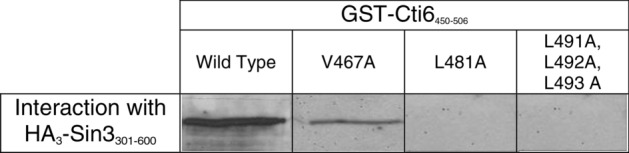
In vitro interaction of GST-Cti6 mutant variants and HA_3_-Sin3 (PAH2). GST-Cti6_450-506_ comprising CSID wild type and missense variants (plasmids pRAR47, pRAR50, pRAR51 and pRAR52) were comparatively analyzed for interaction with HA-tagged Sin3_301-600_ (PAH2) expressed in *S. cerevisiae* (plasmid pYJ91)

Expression plasmids encoding GST fusions of mutagenized Cti6 were synthesized in *E. coli*, bound to GSH-Sepharose and incubated with a protein extract from *S. cerevisiae* containing HA_3_-Sin3_301–600_. GST-Cti6_450–506_ representing the minimal wild-type interaction domain was used as a positive control; GST without fusion protein was used as a negative control. As shown in Fig. 5, Cti6_450–506_ variant V467A is still able to mediate interaction with PAH2, although a weakened interaction signal was obtained. In contrast, the other two mutational variants (Cti6_450–506_ L481A and Cti6_450–506_ L491A L492A L493A) were completely defective for interaction with PAH2 of Sin3.

It can be summarized that leucine residues at amino acid positions 481, 491, 492 and 493 in CSID have a pivotal role in interaction with Sin3. However, it cannot be excluded that a single residue among L491, L492 and L493 is more important for interaction than the remaining leucine residues since all amino acids were replaced simultaneously.

**Fig. 6:**
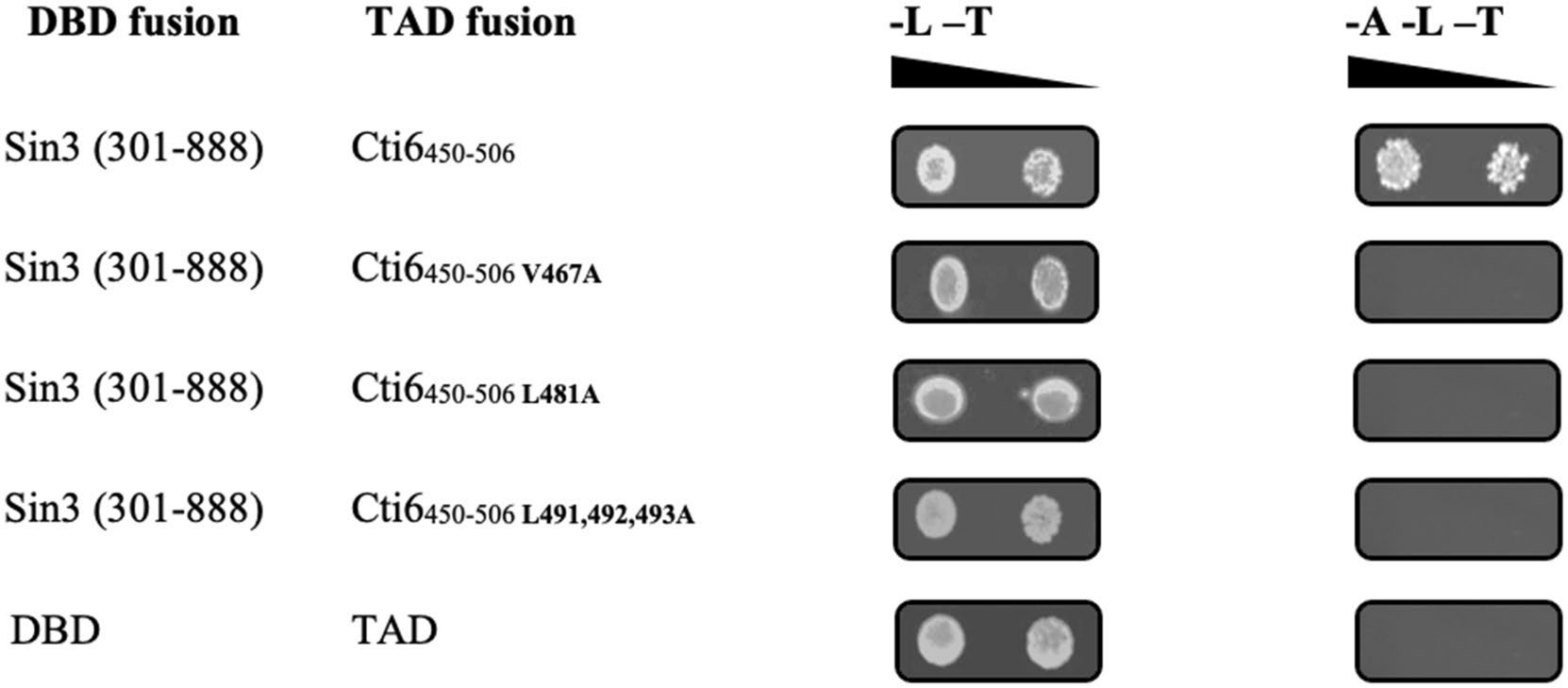
Mutational analysis of Cti6-Sin3 interaction using two hybrid analyses. The Gal4 DNA-binding domain (DBD) was fused with a Sin3 fragment comprising PAH2 to give plasmid pJW50 (aa 301–888). Correspondingly, Gal4 transcriptional-activation domain (TAD) was fused with Cti6_450-506_ wild-type and mutant variants to give pRAR49 (wild type), pRAR65 (V467A), pRAR66 (L481A) and pRAR67 (L491, 492, 493A). As a negative control, empty vectors pGAD-C1 and pGBD-C1 were used. DBD and TAD pairs of fusion plasmids (selection markers: *TRP1* and *LEU2*, respectively) were co-transformed into strain PJ69-4A, containing a *GAL2-ADE2* fusion that allows growth in the absence of adenine when a functional Gal4 activator is reconstituted. Selection plates (-L -T and -L -T -A; absence of leucine, tryptophan and adenine) were incubated for 48 h. Sequence of the mutagenized Cti6 domain (residues 466–493): FVEKVDTIYNGYNESLSMMDDLTRELLLW. Amino acids that were replaced by alanine are underlined

To assay for interaction of Cti6 wild type and missense variants with Sin3 in vivo, we fused Cti6 residues 450–506 with the Gal4 activation domain. The resulting plasmids were co-transformed into strain PJ69-4A with a plasmid encoding Sin3 residues 301–888 (comprising PAH2 and PAH3) fused behind DNA-binding domain of Gal4. In the case of Cti6–Sin3 interaction, the resulting hybrid constructs should be able to activate the Gal4-dependent *GAL2-ADE2* reporter gene which restores growth on adenine-free medium when a functional Gal4 is reconstituted by interaction of the fused proteins. In contrast to the wild-type minimal interaction domain (Cti6_450-506_), Cti6 variants V467A, L481A and L491A L492A L493A were unable to activate the reporter gene (Fig. 6), confirming that these Cti6 variants indeed fail to bind to PAH2 of Sin3.

### The Cyc8-Tup1 co-repressor targets Cti6 protein

**Fig. 7:**
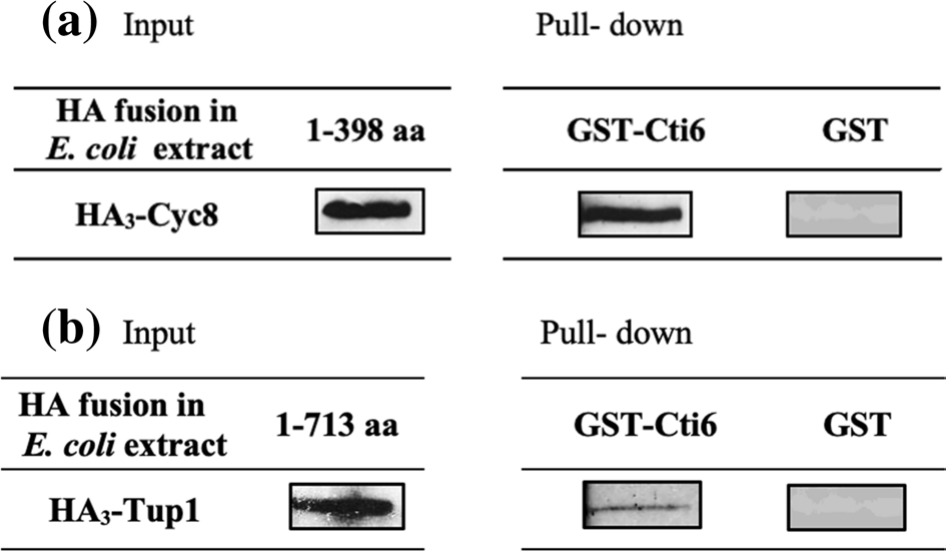
In vitro interaction of Cti6 with Cyc8 and Tup1 individually shown by affinity chromatography. **a** Full length of Cti6 was fused with GST, immobilized on GSH Sepharose and incubated with protein extract from *E. coli* (Strain BL21) containing HA_3_-Cyc8 (plasmid pFK77; 10 TPR motifs). **b** Bacterially synthesized HA-Tup1 (*E. coli* strain BL21, plasmid pRAR110) was incubated with GST-Cti6 (full-length fusion protein) bound to GSH Sepharose. GST-Cti6 fusion is encoded by plasmid pRAR3 (aa 1–506), GST vector was used as a negative control. Extracts containing 75 µg of total protein were analyzed for the input control. To achieve comparable amounts of HA-Cyc8 and HA-Tup1 for the interaction assay, total protein was adjusted accordingly

It has been previously reported that Cti6 directly interacts in vivo and in vitro with the co-repressor Cyc8 (Conlan and Tzamarias 2001; Papamichos-Chronakis et al. 2002). This finding could be confirmed by our studies, again using GST pull-down assays. GST-Cti6 immobilized on GSH Sepharose could associate in vitro with recombinant HA-tagged Cyc8 synthesized in *E. coli* in the absence of additional yeast proteins (Fig. 7a). Furthermore, we also tested for a direct interaction between Cti6 and Tup1. GST-Cti6 full-length fusion was incubated with *E. coli* extract (strain BL21) containing HA_3_-tagged proteins Tup1 protein. As can be seen in Fig. 7b, Cti6 could indeed directly bind also to Tup1.

### CSID interacts with TPR motifs of Cyc8

**Fig. 8:**
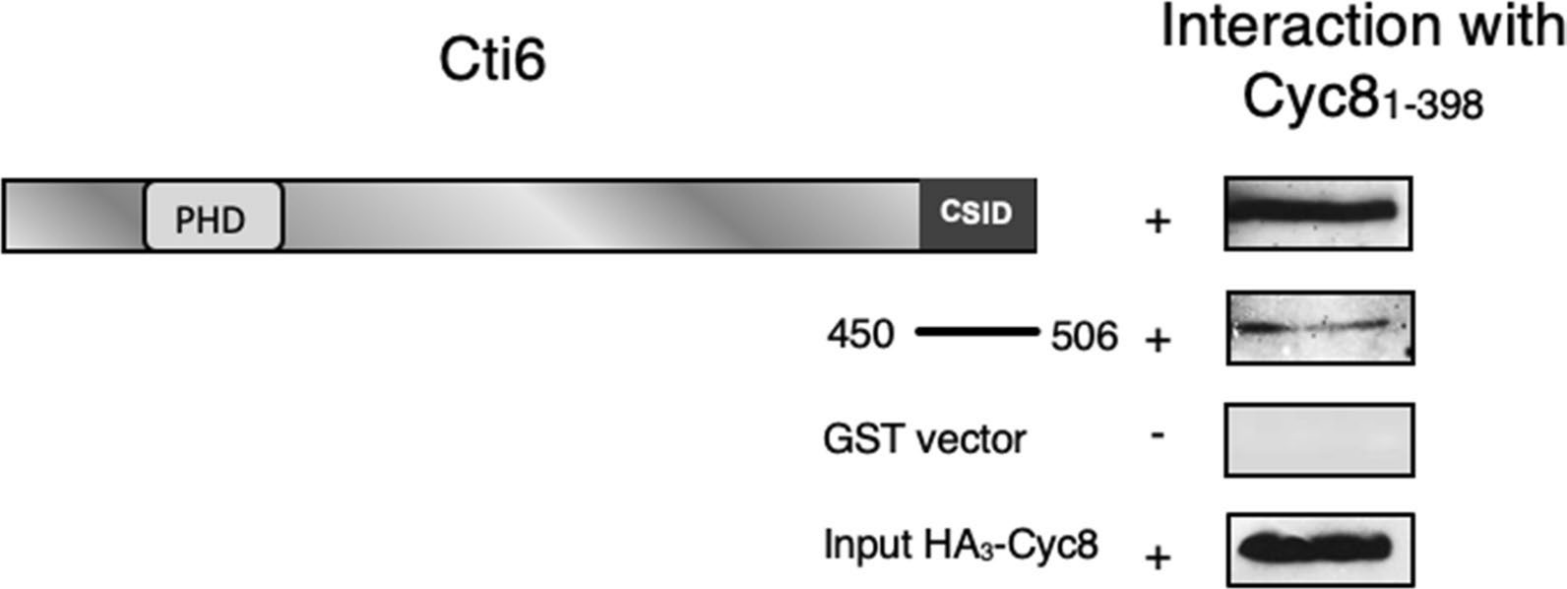
In vitro interaction between length variant GST-Cti6_450-506_ and TPR domains of Cyc8. Length variant GST-Cti6_450–506_ was immobilized on GSH Sepharose and incubated with protein extract from *E. coli* (Strain BL21), containing HA_3_-Cyc8 (plasmid pFK77; 10 TPR motifs). GST-Cti6 fusions are encoded by plasmids pRAR3 (aa 1–506) and pRAR47 (aa 450–506), respectively. GST vector was used as a negative control. Input control is shown at the bottom of the figure (20% of protein used for the interaction assay)

Since previous studies have shown binding of Sin3 and Cyc8 to the same domain of the Opi1 repressor (OSID; Jäschke et al. 2011; Wagner et al. 2001), it is likely (although not inevitable) that pleiotropic co-repressors contact a common interaction domain also in other repressors. To confirm the assumption of Cyc8 being important for Cti6-mediated gene repression, it was interesting to investigate whether the Cti6–Sin3 interaction domain can also interact with Cyc8. Therefore, in vitro interaction of GST-Cti6 (aa 450–506) with HA-tagged Cyc8_1-398_ has been assayed. As shown in Fig. 8, the minimal Cti6–Sin3 interaction domain (CSID; aa 450–506) was also able to interact with the TPR motifs of Cyc8.

### Sin3 is recruited to iron-responsive elements (IRE) containing promoters by interacting directly with Cti6

The functional relationship between Cti6 and Sin3 co-repressor led us to the hypothesis that Sin3 might be present at Cti6 target genes. Chromatin immunoprecipitation assay (ChIP) was employed to monitor directly the occupancy of particular promoters by both regulatory proteins. A growing body of data points to the significant role of Cti6 in iron metabolism (Puig et al. 2004; Crisp et al. 2006). Therefore, two structural genes (*SMF3* and *RNR3*) have been selected as targets for Cti6 regulation. *SMF3* is a member of the Nramp family of divalent metal transporter (Portnoy et al. 2002), which transports iron from the vacuole under iron-limited conditions (Philpott and Protchenko 2008; Kaplan and Kaplan 2009). *RNR3* catalyzes the rate-limiting step in the synthesis of deoxyribonucleotides, playing a major iron-dependent role in DNA replication and repair (Stubbe 2003).

**Fig. 9:**
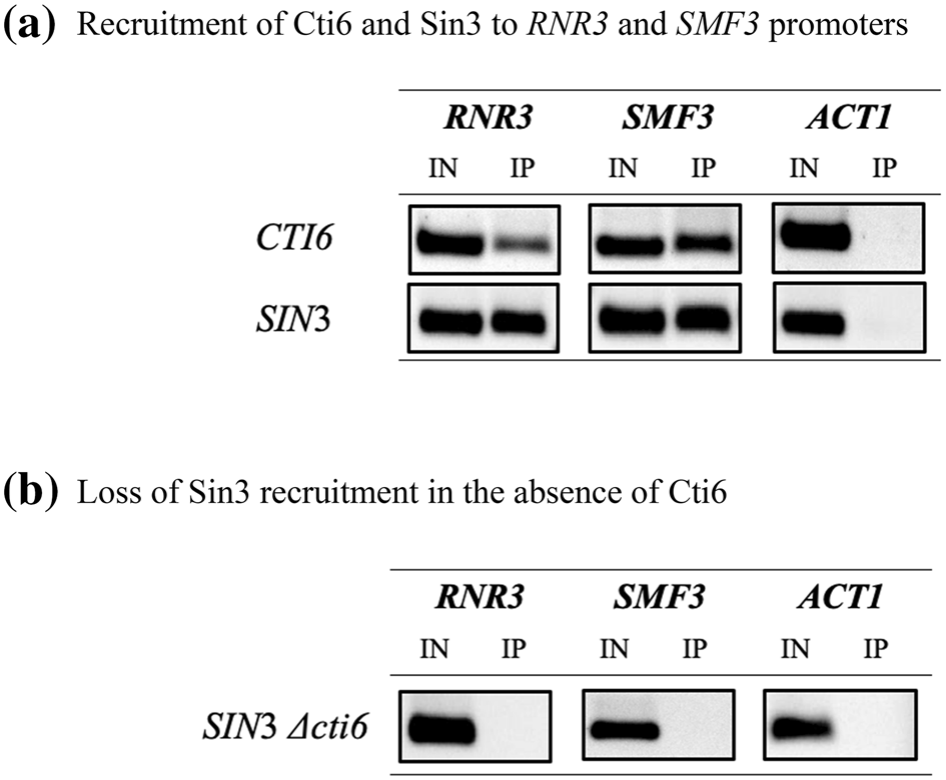
Sin3 recruitment to IRE-containing promoters. Cti6-dependent recruitment of Sin3 co-repressor to IRE-containing promoter regions of *RNR3* and *SMF3* shown by chromatin immunoprecipitation. Strains RAY1 (contains a His-tagged variant of *CTI6* at its natural chromosomal position), RAY3 (isogenic *cti6* deletion mutant) and FKY11 (contains a His-tagged variant of *Sin3* at its natural chromosomal position) were cultivated to the exponential growth phase under repression (+ 100 µM Fe +). After shearing of chromatin, binding to His-Tag Dynabeads® and elution, promoter fragments were analyzed by end-point PCR (**a**) recruitment of Cti6 and Sin3 to *RNR3* and *SMF3* promoters (**b**) loss of Sin3 recruitment in the absence of Cti6, using specific primers for *RNR3, SMF3* and *ACT1* (negative control). PCR products were obtained after 29 amplification cycles and then separated by electrophoresis on a 2% agarose gel. *IN* input control of total chromatin fragments, *IP* analysis of samples obtained by affinity purification

Accordingly, we have first examined the existence of Cti6 at promoters of both target genes under repressive conditions (+ 100 µM Fe +). This is achieved by constructing a strain (RAY1) encoding an epitope-tagged Cti6-HA variant which was subsequently investigated by ChIP analysis. A strain encoding an epitope-tagged variant of Sin3 (Sin3-HA; FKH11) was kindly provided by F. Kliewe (Kliewe et al. 2020). As can be clearly seen in Fig. 9a, both HA-tagged Cti6 and Sin3 proteins were tethered at *SMF3* and *RNR3* promoters under repression in wild-type cells, implicating a role of both regulators for expression of these genes. In vitro experiments have shown Sin3 recruitment by Cti6. Therefore, we further tested whether Sin3 recruitment depends on the function of the Cti6 protein. A *CTI6* gene deletion was introduced into strain FKH11 which encodes epitope-tagged Sin3 (RAY3; Sin3-HA *Δcti6*) and then Sin3 recruitment to target genes was investigated again. As is apparent from Fig. 9b, Sin3 failed to bind to both promoters in the *Δcti6* mutant strain although Sin3 was detected in the presence of an intact copy of Cti6. Thus, Sin3 co-repressor can efficiently be recruited to gene promoters regulated by iron when the Cti6 protein is present.

## Discussion

Cti6 was first identified as Cyc8-Tup1 interacting factor (Papamichos-Chronakis et al. 2000; Conlan and Tzamarias 2001). Additionally, it is known as a subunit of the Rpd3L histone deacetylase complex (Carrozza et al. 2005) responsible for the deacetylation of lysine residues at the N-terminal part of core histones. Histone deacetylation gives a tag for epigenetic repression and plays an important role in transcriptional regulation. Two independent reports have described the association of Cti6 with both Sin3 (Puig et al. 2004) and Cyc8/Tup1 (Papamichos-Chronakis et al. 2002).

But yet no reports investigated more specifically which domain in Cti6 is required for recruitment of the targeted co-repressors. In addition, no reports have explored the domains within Sin3 co-repressor responsible for this interaction. To deduce the features in charge of the function and regulation of co-repressors Sin3, Cyc8 and Tup1 in cells, it is crucial to elucidate a physical map of interacting domains within co-repressors and Cti6 as well. Former reports proposed a functional association of Cti6 with the Rpd3-Sin3–HDAC complex (Ho et al. 2002; Puig et al. 2004) while mapping of its Sin3 binding site has not yet been performed. So, the findings in this work are the first one to decipher such missing features.

In this report, we have clearly confirmed that Cti6 directly interacts with the pleiotropic co-repressor Sin3, indicating that the pathway-specific protein Cti6 executes its function by recruiting the general co-repressor Sin3. Data presented here also indicate that a single domain of yeast protein Cti6 (CSID; aa 450–506) is able to interact with the PAH2 of co-repressor Sin3. No interaction has been found within the C-terminus of Sin3 which could be attributed to the fact that the C-terminal region of Sin3 is associated with complex subunits, such as Sap30, Pho23, etc., which render it inaccessible to other proteins. In addition, the recruitment of HDACs is brought about by interactions with regions in which PAH3 and PAH4 are located (HDACs Rpd3, Hda1 and Hos1 all bind PAH4; Grigat et al. 2012). The "Yeast two-hybrid" system has been used to confirm the examined interactions in vivo*.* It could be affirmed that PAH2 of Sin3 is sufficient for mediating interaction with Cti6. This finding agrees partially with the in vitro mapping results giving evidence that PAH1 and PAH2 of Sin3 can bind to Cti6. In contrast, two-hybrid fusion constructs containing Gal4_TAD_-Cti6_450–506_ (Cti6-Sin3 interaction domain, CSID) and Gal4_DBD_-Sin3_1–300_ (PAH1) could not activate the *GAL2-ADE2* reporter gene which can be attributed to problems with protein folding of Sin3 partial domains under in vivo conditions.

Site-directed mutagenesis of selected hydrophobic residues (V467A, L481A and L491A L492A L493A) has been used to perform a mutational analysis of CSID. While Cti6 interaction with Sin3 appeared to be still functional with a mutated valine (V467A) in vitro (GST pull-down assay), all tested Cti6 variants were defective for the interaction with Sin3 in vivo (two-hybrid assay), indicating that these positions are indispensable for Cti6 binding to Sin3. We suggest that variant V467A may show some residual function which allows interaction in vitro as a result of increased protein amount but is not sufficiently functional in vivo. Although the plant homeodomain (PHD) of Cti6 has an important role for *GAL1* transcriptional activation (Papamichos-Chronakis et al. 2002) and for growth under conditions of iron scarcity as well, our mapping studies demonstrated that this domain is dispensable for its interaction with Sin3.

To obtain further insights into CSID features, we have tested its ability to recruit Cyc8 as well. Interestingly, CSID not only binds to PAH2 of Sin3 but also interacts with TPR motifs of co-repressor Cyc8. Cti6 has been shown to participate in the interaction of the Cyc8-Tup1 co-repressor with the Gcn5-containing SAGA histone acetyltransferase complex (Puig et al. 2002). Moreover, it has been earlier described that amino acid residues 409–506 of Cti6 mediate the interaction with Cyc8 (Papamichos-Chronakis et al. 2002). In this work, we could show that the even shorter CSID (residues 450–506) is able to mediate the interaction with Cyc8_1-398_ (containing only the TPR motifs). In summary, we provide evidence that CSID may be involved in a second pathway of repression acting in parallel to Sin3. Interaction of two co-repressor complexes with a single specific repressor may be the exception but not a general rule, at least in yeast. Besides Cti6, previous reports substantiated that yeast repressor Opi1 being responsible for repression of phospholipid biosynthetic genes contacts Sin3 and Cyc8/Tup1 via a single domain (Jäschke et al. 2011; Wagner et al. 2001). Binding of CSID to PAH and TPR motifs suggests that both interaction modules should be able to contact related targets. Moreover, we also proved the direct interaction of Cti6 with Tup1.

In this work, a versatile in vivo repressor test system was established which allows to confirm a protein as a transcriptional repressor, using a variant strategy initially described by Kadosh and Struhl (1997). We could confirm that Cti6 is able to repress transcription when fused to the DNA-binding domain of LexA. As described by Kadosh and Struhl (1997), amino acids 508–836 of repressor Ume6 contain the Sin3 interaction domain which is sufficient to cause gene repression (decrease of gene expression to approx. 17%) but only in the presence of a functional Sin3 co-repressor.

Thus, expression of truncated variants of lexA-repressor fusion proteins in a suitable reporter strain can be exploited for mapping of repression domains. In this report we showed that Cti6 fused to lexA could reduce the expression of the lexA-dependent reporter gene as efficient as Ume6 (decrease of gene expression to approx. 14%). We hypothesize that Cti6 mediates this repression through recruitment of co-repressors Sin3 and Cyc8-Tup1. In future work, construction of *SIN3* and *CYC8* gene deletions mutants could provide evidence for the relative importance of these co-repressors for reduction of gene expression. It is expected that the repression effect is abolished when co-repressors are absent. Previously, Puig et al. (2004) demonstrated that Cti6-mediated transcriptional repression was substantially alleviated in the absence of the histone deacetylase Rpd3.

To provide further mechanistic insight on the function of Cti6 and its association with Sin3 co-repressor, it is crucial to elucidate the role of proteins that function with this pleiotropic co-repressor, such as Cti6. Two structural genes (*RNR3* and *SMF3*) have been selected as targets for Cti6 regulation. Both structural genes are playing an important role in iron metabolism (Puig et al. 2004; Stubbe 2003). In this work, a functional association of Cti6 with Sin3 co-repressor could be proved by chromatin immunoprecipitation. The in vivo occupancy of *RNR3* and *SMF3* promoters by Cti6 and Sin3 has been individually monitored under repression. Furthermore, we assayed the occupancy of Sin3 on the respective promoters by constructing a strain lacking Cti6. Results of our ChIP analysis confirmed that Sin3 is no longer present at these promoters in the absence of Cti6 (*Δcti6* mutant), indicating that Sin3 recruitment depends on Cti6. These results are in agreement with data of Puig et al. (2004) who demonstrated that *SMF3* and *RNR3* were up-regulated in a *cti6* mutant. This allows the assumption that both pleiotropic co-repressors Sin3 and Cyc8/Tup1 work together via the interaction with Cti6 leading to gene repression.

Cti6 is recruited to iron-responsive promoters in an Aft1-dependent manner and mediates the expression of *ARN1* under heme-deficient conditions (Crisp et al. 2006). As Cti6 is not a DNA-binding protein, the question arises how Cti6 tethers to the structural gene promoters and mediates Sin3 recruiting. We propose two different models concerning each structural gene. Ribonucleotide–diphosphate reductase (encoded by *RNR3*) catalyzes the rate-limiting step in deoxyribonucleotide synthesis and plays an essential iron-dependent role in DNA replication and repair (Stubbe 2003). *RNR3* repression is mediated by upstream repression sequences (URS) designated as damage-responsive elements (DREs) that serve as binding sites for the sequence-specific DNA-binding protein Rfx1 (Huang et al. 1998).

Accordingly, it could be suggested that under repressing conditions Cti6 binding to the *RNR3* promoter occurs in this order, Rfx1 binds to the structural gene promoter, allowing entry of Cti6 which mediates repression through Sin3 and Cyc8/Tup1 recruitment. This assumption is strongly supported by the findings of Bing and Joseph (2001) who could show that *RNR3* repression is mediated by Cyc8, Tup1 and Rfx1, causing changes in nucleosome positioning and chromatin structure. Therefore, the supposed interaction between Cti6 and Rfx1 should be investigated in the future. It should be emphasized that direct in vitro interaction between Rfx1 and Cyc8 has been confirmed in independent work (Huang et al.^1998^; Zhang and Reese 2005).

**Fig. 10:**
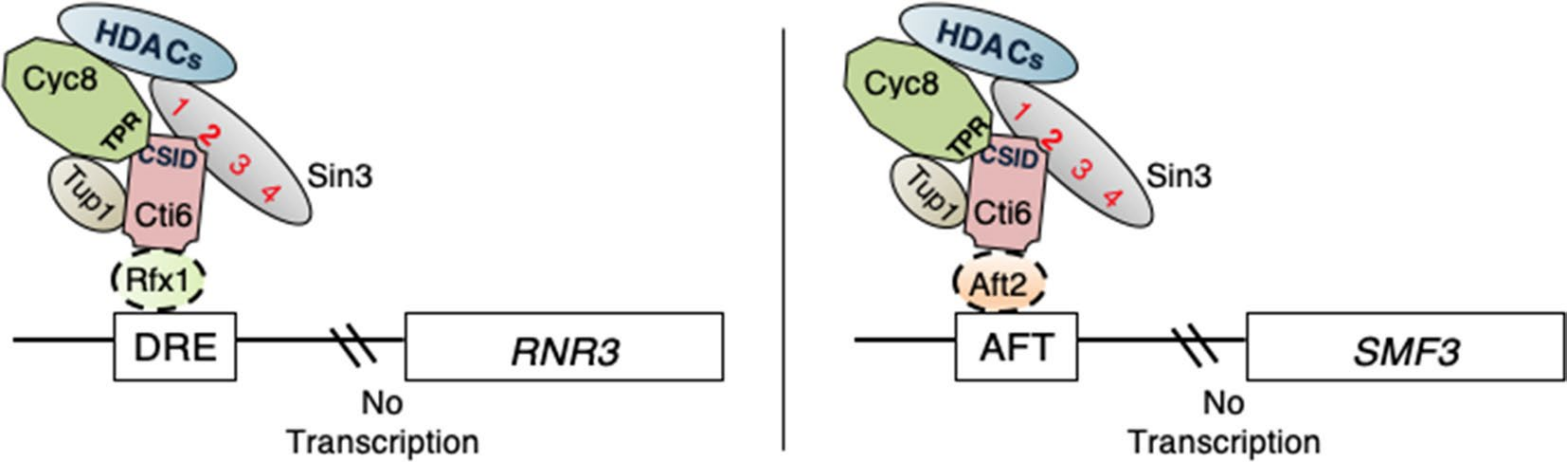
A schematic representation of two hypothetical models of Sin3 dependent Cti6 recruitment. *RNR*3 and *SMF*3 are iron-regulated structural genes. Under repression (+ 100 µM Fe +), Anchoring of Cti6 on the DRE region of *RNR3* and AFT of *SMF3* promoters occurs. This may be implemented via Cti6 recruitment by Rfx1 and Aft2 (in dashed oval shape as speculative scenario), respectively. Then, Cti6 recruits Sin3 co-repressor through the interaction between PAH2 and CSID which triggers a conformational reorganization, bringing HDACs into action preventing transcription of the respective genes. The existence of Cyc8 and Tup1 should be considered through CSID/TPR and Cti6/Tup1 interaction. DRE, damage-responsive elements; AFT, activator of Fe transcription; CSID, Cti6-Sin3 interaction domain; TPR, tetratricopeptide repeat. Numbers 1, 2, 3 and 4 within the Sin3 oval indicate paired amphipathic helices PAH1, 2, 3 and 4

A related, but slightly different situation may be effective in the case of *SMF3* whose upstream region possesses a pair of Aft consensus binding sequences (Portnoy et al. 2002). The DNA-binding protein Aft2 (Blaiseau et al. 2001; Rutherford et al. 2003) binds and activates the transcription of *SMF3* (Courel et al. 2005). Interestingly, *SMF3* is not only induced under conditions of iron starvation (Portnoy et al. 2000), but also up-regulated in *cti6* mutant (Puig et al. 2004). Thence, as Cti6 is not a DNA-binding protein and can be detected on *SMF3* gene promoter by ChIP assay, we assume that Cti6 was recruited by Aft2 under repression (+ 100 µM Fe +) inhibiting *SMF3* expression by recruiting the pleiotropic co-repressors Sin3 and Cyc8/Tup1. Further work should investigate the possible direct or indirect interaction between Cti6 and Aft2 under both repression and de-repression conditions. Both hypothetical models are summarized in Fig. 10. Taken together, our results unraveled novel structural insights into the multifunctional regulator Cti6 as we could precisely identify the critically important domain within Cti6 that directly recruits not only Sin3 (via PAH2 domain) but also Cyc8 (via TPR motifs) co-repressors. In addition, our findings provide unprecedented evidence of Sin3 in vivo binding on promoters of genes involved in iron response only in the presence of Cti6. In conclusion, this work has addressed the functional role of member of Rpd3L histone deacetylase complex in yeast model supporting the evidence that one domain (CSID) is implicated with multiple co-repressor complexes Sin3 and Cyc8-Tup1 in related subtle molecular events.

## Electronic supplementary material

Below is the link to the electronic supplementary material.

Supplementary file1 (DOCX 41 kb)
