# Supplementary material for: Functional analysis of Cti6 core domain responsible for recruitment of epigenetic regulators Sin3, Cyc8 and Tup1
Source: Curr Genet. 2020 Sep 26;66(6):1191–203. doi: 10.1007/s00294-020-01109-4 (PMC7599196; doi:10.1007/s00294-020-01109-4)
Supplement: Supplementary file 1 — Supplementary file1 (DOCX 41 kb) [file 294_2020_1109_MOESM1_ESM.docx]

**Supporting Online Table S1:**

**a) Strains of *Saccharomyces cerevisiae* used by Aref and Schüller**

| **Strain** | **Genotype** |
| --- | --- |
| C13-ABY.S86 | *MAT*α *ura3 leu2 pra1 prb1 prc1 cps1* |
| PJ 69- 4 A | *MATα trp1-901 leu2-3,112 ura3-53 his3- 200 ∆gal4 ∆gal80 GAL2-ADE2 lys2 :: GAL1-HIS3 met2::GAL7-lacZ* |
| FKY11 | *MATα ura3 leu2 pra1 prb1 prc1 cps1 SIN3-HA_3_::kanMX* |
| RAY1 | *MATα ura3 leu2 pra1 prb1 prc1 cps1 CTI6-HA_3_::kanMX* |
| RAY3 | *MATα ura3 leu2 pra1 prb1 prc1 cps1 SIN3-HA_3_ :: kanMX Δ cti6 :: LEU2* |
| NKTS | *MATα ura3 leu2 his3 trp1 CYC1-lacZ::URA3* |
| RTS + lexA | *MATα ura3 leu2 his3 trp1 lexA_OP_ CYC1- lacZ::URA3* |

**b) Plasmids constructed and used by Aref and Schüller**

| **Plasmid** | **Genotype** |
| --- | --- |
| pCW117 | 2μm *URA3 MET25*_Pr_-HA_3_-*SIN3-CYC1*_Ter_ (full length); Wagner *et al.,* 2001 |
| pSW11 | tet_Pr/Op_-HA_3_-*SIN3* (full length); Grigat *et al.,* 2012 |
| pRAR110 | tet_Pr/Op_-HA_3_-*TUP*1 (full length) |
| pFK77 | tet_Pr/Op_-HA_3_-*CYC8* (10 TPR Motifs, aa 1-398) |
| pCW83 | MET25_PRO_-HA_3_-*SIN3*_1-300_ *CYC1*_TER_2μm *URA*3 Jäschke *et al.* 2011 |
| pYJ91 | MET25_PRO_-HA_3_-*SIN3*_301-600_ *CYC1*_TER_2μm *URA*3 Jäschke *et al.* 2011 |
| pYJ90 | MET25_PRO_-HA_3_-*SIN3*_601-950_ *CYC1*_TER_2μm *URA*3 Jäschke *et al.* 2011 |
| pYJ89 | MET25_PRO_-HA_3_-*SIN3*_801-1100_*CYC1*_TER_2μm *URA*3 Jäschke *et al.* 2011 |
| pMP20 | MET25_PRO_-HA_3_-*SIN3*_1101-1536_*CYC1*_TER_2μm *URA*3 Jäschke *et al.* 2011 |
| pJW6 | ADH1_PRO_*-GAL4*_DBD_*-SIN3*_1-300_*-ADH1*_TER_*2μm TRP1* Wittmann, 2000 |
| pJW50 | ADH1_PRO_*-GAL4*_DBD_*- SIN3*_301-888_*-ADH1*_TER_ *2μmTRP1* Wittmann, 2000 |
| pRAR3 | tac_Pr/Op_-GST-*CTI*6 (aa 1-506, full length) |
| pRAR10 | tac_Pr/Op_-GST-*CTI*6 (aa 1-196) |
| pRAR11 | tac_Pr/Op_-GST-*CTI*6 (aa 197-506) |
| pRAR14 | tac_Pr/Op_-GST-*CTI*6 (aa 241-350) |
| pRAR15 | tac_Pr/Op_-GST-*CTI*6 (aa 351-506) |
| pRAR30 | tac_Pr/Op_-GST-*CTI*6 (aa 351-429) |
| pRAR31 | tac_Pr/Op_-GST-*CTI*6 (aa 430-506) |
| pRAR47 | tac_Pr/Op_-GST-*CTI*6 (aa 450-506) |
| pRAR20 | ADH1_PRO_-*GAL*4_TAD_-*CTI*6_(aa 351-506)_-*ADH*1_TER_ 2μm *TRP*1 |
| pRAR37 | ADH1_PRO_-*GAL*4_TAD_-*CTI*6_(aa 430-506)-_*ADH*1_TER_ 2μm *TRP*1 |
| pRAR49 | ADH1_PRO_-*GAL*4_TAD_-*CTI*6_(aa 450-506)_-*ADH*1_TER_ 2μm *TRP*1 |
| pRAR50 | tac_Pr/Op_-GST-*CTI*6_(aa 450-506)_ V467A, (Mut. - PCR pRAR47) |
| pRAR51 | tac_Pr/Op_-GST-*CTI*6_(aa 459-506)_ L481A, (Mut. - PCR pRAR47) |
| pRAR52 | tac_Pr/Op_-GST-*CTI*6_(aa 459-506)_ L491,492,493A, (Mut. - PCR pRAR47) |
| pRT-lexA | *MET25*-l*exA*_DBD_-NLS |
| pRAR27 | *MET25*-HA_3_-lexA_DBD_-NLS-*CTI*6 (aa 1-506, full length) |
| pRAR65 | *ADH*1_PRO_-*GAL*4_TAD_-*CTI*6_450-506_ [V467A]-*ADH*1_TER_2μm *TRP*1 |
| pRAR66 | *ADH*1_PRO_-*GAL*4_TAD_-*CTI*6_450-506_ [L481A]-*ADH*1_TER_ 2μm *TRP*1 |
| pRAR67 | *ADH*1_PRO_-*GAL*4_TAD_-*CTI*6_450-506_ [L491,492,493A]-*ADH*1_TER_ 2μm *TRP*1 |
| pRAR81 | *ΔCTI6::LEU2* |

aa, amino acids; Pr, promoter; Op, operator; Ter, terminator; Mut, mutation; tetratricopeptide repeats (TPR)

**c) Table of oligonucleotides used by Aref and Schüller**

**(Construction of expression plasmids for GST- tagged *Cti6* (full length and length variants); PCR primers for *Cti6* strain modifications, and *SMF3* and *RNR3* for ChIP analyses)**

| Name | Gene | Position | Sequence (5'-3') |  |  |
| --- | --- | --- | --- | --- | --- |
| CTI6 START *Bgl*ll | *CTI*6 | +1 /+20 | gact**agatct**ATGGAATCGACAGCAATAGT |  |  |
| CTI6 STOP *Xho*l | *CTI*6 | +1521/+1502 | gact**ctcgag**TTATTGAATGGCATTAGTGT |  |  |
| CTI6 5F aa 197 *Bgl*II | *CTI*6 | +591/+610 | gatc**agatct**GCTGTAGCATTAGCCAAGGA |  |  |
| CTI6 5F aa 230 *BamH*l | *CTI*6 | +690/+709 | gatc**ggatcc**GGAGATAGTGATAAGAAGCT |  |  |
| CTI6 3R aa 350 *Hind*III | *CTI*6 | +1050/+1031 | gatc**aagctt**TCAAGTGTCTTGCGCCGAACCCG |  |  |
| CTI6 5F aa 450 *BamH*l | *CTI*6 | +1350/+1369 | gatc**ggatcc**GATCAGTCTGATCGAGAGGA |  |  |
| CTI6 3R aa 429 *Xho*l | *CTI*6 | +1287/1268 | gact**ctcgag**TCACATTTCGTTTAATGAGGTCC |  |  |
| CTI6 5F aa 430 *BamH*l | *CTI*6 | +1290/1309 | gact**ggatcc**AGAAGAAGGGTATCCGCTAT |  |  |
| CTI6 3R aa 350 *Xho*l | *CTI*6 | +1050/+1031 | gatc**ctcgag**TCAAGTGTCTTGCGCCGAACCCG |  |  |
| CTI6 5F aa 351 *BamH*l | *CTI*6 | +1053/+1072 | gatc**ggatcc**GAGAAAACTGATGAACCCAT |  |  |
| CTI6 3R aa 506 *Cla*l | *CTI*6 | +1521/+1502 | gact**atcgat**TTATTGAATGGCATTAGTGTT |  |  |
| CTI6 5F aa 241 *BamH*l | *CTI*6 | +723/+742 | gatc**ggatcc**AGGGCAACTTTCATGGCAAG |  |  |
| CTI6 5F V467A | *CTI*6 | +1372/+1421 | GTACGATTCGTGGAAAACCAGCATTTC**GCA**GAAAAAGTTGATACGATTTA |  |  |
| CTI6 3R V467A | *CTI*6 | +1421/+1372 | TAAATCGTATCAACTTTTTCTGCGAAA**TGC**TGGTTTTCCACGAATCGTAC |  |  |
| CTI6 5F L481A | *CTI*6 | +1412/+1471 | ATACGATTTACAACGGTTATAATGAAAGT**GCA**TCAATGATGGACGACCTGACTAGAGAGT |  |  |
| CTI6 3R L481A | *CTI*6 | +1471/+1412 | ACTCTCTAGTCAGGTCGTCCATCATTGA**TGC**ACTTTCATTATAACCGTTGTAAATCGTAT |  |  |
| CTI6 5F L491,492, 493A | *CTI*6 | +1473/+1532 | TATCAATGATGGACGACCTGACTAGAGAG**GCAGCAGCA**TGGGAGAAAAAATATTCAAATA |  |  |
| CTI6 3R L491,492, 493A | *CTI*6 | +1532/+1473 | TATTTGAATATTTTTTCTCCCA**TGCTGCTGC**CTCTCTAGTCAGGTCGTCCATCATTGATA |  |  |
| CTI6-Ver | *CTI*6 | +1270/+1289 | ACCTCATTAAACGAAATGAG |  |  |
| CTI6 FOR | *CTI*6 | +1474/+1518 | CTACTATGGGAGAAAAAATATTCAAATAACACTAATGCCATTCAAtcccaccaccatcatcatcac |  |  |
| CTI6 REV | *CTI*6 | +1711/+1667 | ATGTGCTAGTCCATTAGATGTTTGGATCCTTAATCATCGTGCCAactatagggagaccggcagat |  |  |
| CTI6-5F-*EcoR*l | *CTI*6 | -453/-434 | gactgaaTTCACCCACTTCTGGCACTT |  |  |
| CTI6-5F-*BamH*l | *CTI*6 | -50/-69 | gactggatcCGCAAAGTAAACTTCGAAGG |  |  |
| CTI6-3F-*Xba*l | *CTI*6 | +1568/+1587 | gacttctaGAGGCGAACAAACACATCTA |  |  |
| CTI6-3F-*Hindl*ll | *CTI*6 | +1982/+1963 | GTTAAAACTCTACGCAAACC |  |  |
| CTI6-Ver | *CTI*6 | +2010/+1991 | ATAATCATTATCCAGGGTGC |  |  |
| CTI6-ORF | *CTI*6 | +1241/+1260 | CAGTAAAGCCCAGATTACCC |  |  |
| LEU2-ORF | *LEU*2 | +1024/+1043 | TAGGTGGTTCCAACAGTACC |  |  |
| kanK2 | *kanMX* | +52/+70 | GGGCGACAGTCACATCATGC | |  |
| SMF3 FOR | *SMF*3 | -350/-329 | TGCCCGCCCTGCCTCTCTTCC | | |
| SMF3 REV | *SMF*3 | -80/-99 | TAGTTTGGGTATGACAACTG | | |
| RNR3 FOR | *RNR*3 | -400/-381 | AGCCAAGTTATCTGCCTACG | | |
| RNR3 REV | *RNR*3 | -100/-118 | GAAGGCAAAATAGCAGTGCG | | |

Artificially inserted cleavage sequences for restriction enzymes are shown in **bold**; capital letters represent genuine gene-specific sequences.
